# Plasmonic porous micro- and nano-materials based on Au/Ag nanostructures developed for photothermal cancer therapy: challenges in clinicalization

**DOI:** 10.1039/d3na00763d

**Published:** 2023-11-27

**Authors:** Reza Taheri-Ledari, Fatemeh Ganjali, Simindokht Zarei-Shokat, Reihane Dinmohammadi, Fereshteh Rasouli Asl, Ali Emami, Zahra Sadat Mojtabapour, Zahra Rashvandi, Amir Kashtiaray, Farinaz Jalali, Ali Maleki

**Affiliations:** a Catalysts and Organic Synthesis Research Laboratory, Department of Chemistry, Iran University of Science and Technology Tehran 16846-13114 Iran Rezataheri13661206@gmail.com R_taheri94@alumni.iust.ac.ir maleki@iust.ac.ir +98 2173021584 +98 21 77240640-50

## Abstract

Photothermal therapy (PTT) has developed in recent decades as a relatively safe method for the treatment of cancers. Recently, various species of gold and silver (Au and Ag) nanostructures have been developed and investigated to achieve PTT due to their highly localized surface plasmon resonance (LSPR) effect. Concisely, the collective oscillation of electrons on the surface of Au and Ag nanostructures upon exposure to a specific wavelength (depending on their size and shape) and further plasmonic resonance leads to the heating of the surface of these particles. Hence, porous species can be equipped with tiny plasmonic ingredients that add plasmonic properties to therapeutic cargoes. In this case, a precise review of the recent achievements is very important to figure out to what extent plasmonic photothermal therapy (PPTT) by Au/Ag-based plasmonic porous nanomedicines successfully treated cancers with satisfactory biosafety. Herein, we classify the various species of LSPR-active micro- and nano-materials. Moreover, the routes for the preparation of Ag/Au-plasmonic porous cargoes and related bench assessments are carefully reviewed. Finally, as the main aim of this study, principal requirements for the clinicalization of Ag/Au-plasmonic porous cargoes and their further challenges are discussed, which are critical for specialists in this field.

## Abstract 2

Photothermal therapy (PTT) has developed in recent decades as a relatively safe method for the treatment of cancers.

## Graphical abstract

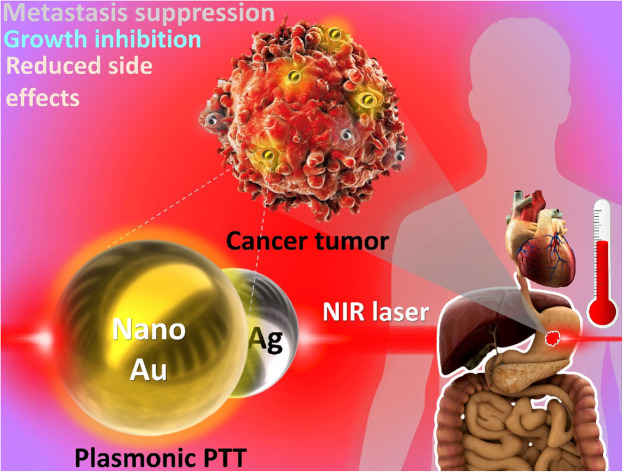

## 1. Introduction

In the fight against cancer, the most challenging goal is considered to be its prevention and treatment.^1^ The most important factor contributing to this may be the rapid spread of cancer cells in the body, which is known as “metastasis” in medical science.^2^ In fact, conventional methods appear to affect the healing process at a slow rate, resulting in the progression of this disease through the fast growth of cancer cells. As a well-known clinical method, chemotherapy has been widely applied, in which several cytotoxic medication doses are successively administered to the patient.^3^ However, chemotherapy is a slow-responding and unsafe treatment method because of its limitations in terms of drug dosage and non-selectivity in targeting cancer cells.^4^ Also, due to the above-mentioned drawbacks, chemotherapy involves a relatively tight therapeutic window compared to novel developed strategies.^5^ Hence, treatment approaches based on safer and faster strategies have been developed, in which various physicochemical properties of tiny-scale materials have been exploited to fight cancer.^1^ In the last decade, many types of micro- and nano-scale materials have been evaluated to develop new treatment programs, such as targeted drug delivery,^6,7^ hyperthermia (HT),^8^ gene delivery,^9^ photodynamic therapy (PDT),^9^ radiotherapy,^10^ photoimmunotherapy (PIT),^11^ and PTT.^12^

**Fig. 1:**
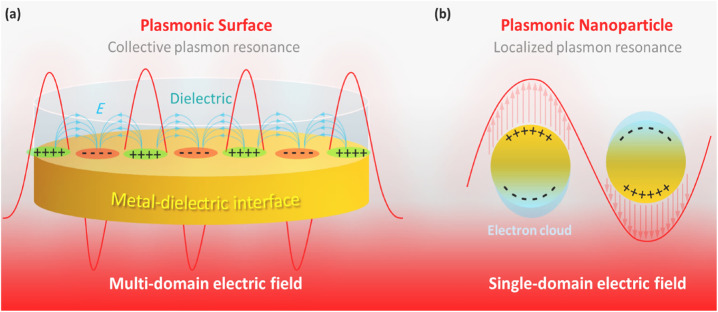
Schematic illustration of (a) collective SPR and (b) localized SPR.

Among the newly developed approaches, PTT has emerged as a suitable alternative to chemotherapy because it does not require the use of a cytotoxic agent. Consequently, the relative side effects are significantly reduced. Concisely, PTT is based on heating through the irradiation of a specific wavelength of the electromagnetic spectrum.^9^ Presently, the surface plasmon effect at metal–dielectric interfaces, as a unique optical property of nanomaterials, is used for PTT-based diagnosis and treatment of cancers.^13^ Through this effect, the electron density is concentrated at specific areas on the metallic surfaces (such as gold and silver) through the longitudinal passage of a particular electromagnetic wavelength.^14^ The localization of these plasmonic-gathered electrons by limiting the dimensions to the nanoscale is called LSPR, which has been considered for PTT applications (Fig. 1a and b).^15^ In fact, the term LSPR refers to the collective oscillation of free electrons on tiny-size metallic nanoparticles (NPs) upon exposure to a specific wavelength (depending on the particle size), which generates heat through the rapid relaxation of the excited electrons. The LSPR effect in gold NPs (as a well-known agent for PTT) leads to absorption at 520 nm in the visible light spectrum, resulting in a red color.^16^ Based on the submitted descriptions, tiny-sized materials that possess the LSPR property are great candidates for PTT applications. Generally, the generation of heat through irradiation at a specific wavelength in plasmonic materials for tumor destruction is called PPTT.

Historically, PPTT was developed by Naomi Halas, who worked on the plasmonic response of gold to infrared waves in 1998.^17^ Hirsch *et al.* conducted one of the first studies on PPTT at an annual conference in biomedical engineering in 2002.^18^ In their report, gold nanoshells with LSPR at 821 nm were synthesized and conjugated to antibodies (Abs) for selective targeting. A near-infrared (NIR) laser was employed to induce plasmonic heating in the gold nanoshells. Generally, depending on the particle size and morphology, the LSPR of the active nanostructures (mainly gold and silver) can be adjusted to the NIR region, which is not absorbed by the skin and can penetrate tissues.^19^ Different Ag/Au NP morphologies, most frequently spheres, rods, and stars, provide special benefits for cancer therapy.^20–22^ For instance, spherical structures require the simplest synthesis and bioconjugation processes, favoring PPTT. Due to their capability to generate second-order resonance, Au nanospheres can be used for PPTT, although they only exhibit substantial absorption in the visible light region. Due to their second-order non-linear optical characteristics, NIR light-generated photons that interact with Au nanospheres combine and transform into new low-energy photons with double the frequency that can resonate with the Au nanospheres. The transverse and longitudinal surface plasmon resonance peaks of Au nanorods (AuNRs) indicate longitudinal and transverse SPR effects, respectively, with the longitudinal light absorption peak in the NIR range. Consequently, when exposed to NIR light, AuNRs may effectively convert light into heat based on the SPR effect. *In vivo* research revealed that AuNRs did not affect liver or kidney function under NIR laser irradiation. Au nanostars exhibit the SPR effect in the NIR light spectrum, which can change their light absorption peak depending on the quantity of star points. The light absorption peak will be red-shifted with an increase in the number of tips. In PPTT, the numerous branches of Au nanostars contribute to a substantially bigger surface area than that of their counterparts. More plasma can resonate with NIR light over the greater surface area, trapping more induced electrons. However, Au nanostars often have a higher hydrodynamic diameter than their counterparts, which may restrict the antitumor effectiveness and impact the cellular absorption efficiency.^23–25^ In 2010, Zahi Fayad exploited the PPTT technique for imaging and studying atherosclerosis plaque by gold NPs.^26^ Subsequently, in 2012, photothermal theranostics (therapeutic plus diagnostic) was introduced by Wang's research team through their bench studies on metal-doped carbon nanotubes (CNTs).^27^ Efforts continued employing several species of materials for PPTT applications, such as graphene and carbon quantum dots,^28^ gold and silver NPs,^29^ palladium,^30^ and copper sulfide and tungsten disulfide.^31^

However, to date, although great achievements have been realized in bench studies, the clinicalization of the developed PPTT methods needs to overcome many challenges. Firstly, the stability and half-life of the PPTT-cargo should be optimized because a high degree of stability leads to long-time circulation in the blood serum and distribution in non-target tissues and further damage. In contrast, low structural stability results in the early extinction of the PPTT-cargo and its rapid degradation.^32^ In many cases, the size of the PPTT-cargo is not small enough to safely pass through the arteries in the kidney, and consequently its degradation in the blood serum is more probable than excretion. Hence, the biodegradability of the designed cargo should be seriously considered.^33^ Another issue is selectivity and high targeting in the delivery of the PPTT-cargo to the tumor site. Obviously, the entry of the injected cargo into the healthy tissues can damage normal cells, resulting in negative side effects. Thus, to induce selectivity in the designed PPTT-cargo, stable conjugation with biologically active ingredients such as Abs, folic acid, and aptamers (Apts) is applied.^34^ To achieve this goal, the architecture of plasmonic materials may require engineering, through which chemically active sites are added to their structure. The efficiency (PPTT performance) per injected dose of the PPTT-cargo should also be satisfactory.

In some cases, the structural engineering of the PPTT-cargo affects their plasmonic content and activity, reducing the total performance of the designed cargo.^35^ Thus, to address this problem, the use of highly porous materials (as hosts) in which a large amount of plasmonic materials can be doped is recommended. Moreover, the biocompatibility and biosafety of the host materials should be precisely investigated.^36^ In addition to all the above-mentioned challenges, the design and preparation of multi-functional cargoes have been widely reported in recent years.^37^ Recent reports have demonstrated amazing results of the simultaneous application of PPTT and chemotherapy.^38^ In these advanced systems, the cytotoxic agent loaded in the host material (polymeric, inorganic, and natural species) is released with high control through the PPTT properties of the host.^39^ Consequently, great synergistic effects have been observed between the chemical targeting and PPTT killing of cancer cells. Also, the incorporation of a large amount of plasmonic materials in the host can result in a dual-application cargo, *i.e.*, PPTT treatment and diagnosis, which is called “theranostics”.^40^ As a substantial tool, highly porous materials have been recently developed for plasmonization by silver and gold nanoparticles (Ag and AuNPs) as the most efficient components with LSPR properties.^41^

The term “plasmonization” is suggested for hybrid systems, including the LSPR-active materials in their structures. Specifically, it is better to distinguish the concepts of “plasmonic” and “plasmonized”, where the former refers to materials that inherently possess the LSPR effect, whereas the latter is related to systems equipped with plasmonic ingredients. Thus far, various species of porous materials have been recognized to be appropriate candidates for doping LSPR materials, including silica,^42^ clay-based tubes and spheres,^43^ metal–organic frameworks (MOFs),^44^ and carbon-based tubes and sheets.^45^ One of the most suitable candidates for PPTT for overcoming the abovementioned challenges is MOFs, a well-known category of inorganic substances based on coordination chemistry.^46–49^ Concisely, MOFs possess huge porosity, making them appropriate hosts for large amounts of plasmonic materials and co-therapeutic agents.

Also, considering the various applications of the LSPR effect of Au and Ag NPs, the electrocatalytic hydrogen evolution activity of Co–Fe-MOF nanosheets (Co–Fe-MOFNs) is dramatically enhanced by the LSPR excitation of AuNRs according to Wang *et al.* This resulted in a more than 4-fold increase in current density at −0.236 V (*vs.* RHE) for the Au/Co–Fe-MOFNs composite under light illumination compared to in the dark.^50^ Examples of chemical changes studied utilizing plasmonics and electrochemistry include nitro-aromatic compounds and diazonium salts. On a silver surface that had been roughened, the LSPR-activated oxidation of *para*-aminothiophenol to produce 4,4′-dimercaptoazobenzene was accomplished effectively. Recently, the plasmonic effect of AuNPs was investigated for water splitting and glucose electrocatalysis. The plasmon-accelerated electrochemical reaction mechanism was postulated based on experimental research.^51^ Plasmonic NPs, following LSPR stimulation, may also speed up electrochemical processes. The direct plasmon-accelerated electrochemical reaction (PAER) on AuNPs was observed using a model reaction system for the electrolysis of glucose. The increased electrocatalysis performance is attributed to the hot charge carriers produced during plasmon decay, as shown by the wavelength- and solution pH-dependent electrochemical oxidation rate and the results of dark-field scattering spectroscopy.^52^ The plasmonic NPs introduced in a recent study with diverse geometries, including dimeric, tetrameric, and multimeric with higher orders, core–satellite architectures, and nano chains, are excellent nanoplatforms for various biological applications due to their tunable optical characteristics. Generally, these plasmonic nano assemblies possess many advantages compared to individual AuNPs, such as stronger electromagnetic field, more functionality, more flexible responsiveness, and wider applications.^53^ In a recent study, a phosphorescent gold(i) complex emitted white light phosphorescence. Here, the molecular packing, which was related to the emission wavelength and intensity, was determined by the Au–Au bonds in the trimeric gold(i) complex, confirming their presence as a prerequisite in the synthesis of novel chromophores. The gold complex was subjected to temperature and mechanical grinding tests, and its long-wavelength peak was eliminated and redshifted, proving that the trimer configuration caused by aurophilic interaction was the primary factor determining the long-wavelength emission of the chromophore.^54^

Generally, tiny-sized materials can act as a supporting layer for embedded plasmonic materials to protect them from rapid degradation in the blood serum. The exterior surface of these materials can also be conjugated with biologically active ingredients for inducing selective function in PPTT-cargo.^55^ Herein, our aim is to present how the PPTT efficiency directly depends on the engineered architecture of the utilized plasmonic composites, focusing on micro- and nano-scale cargo based on Ag and AuNPs. We intend to exclusively investigate the potential of silver- and gold-plasmonic porous cargoes (abbreviated as PPCs) for clinicalization in cancer therapy, and also the possible challenges in achieving this. Hence, this content can provide insight for researchers seeking feasible strategies to bring PPTT methods in the clinic through more powerful ways. Due to their selectivity and non-intrusive properties, nanomaterials with photothermal efficiency have attracted significant interest. In this study, we present many innovative photothermal nanomaterials and their biomedical uses, such as silica materials, CNTs, MOFs, and nanomaterials based on noble metals such as AuNPs, Ag NPs, and Au–Ag. Here, the synthesis, bench assessment, and photothermal conversion characteristics of the aforementioned photothermal NPs are particularly important. Also, the uses of these photothermal nanomaterials are demonstrated and explored. Finally, the challenges and future of photothermal materials in the rapidly developing field of scientific study are discussed.

## 2. A historical overview of PTT and PPTT

**Fig. 2:**
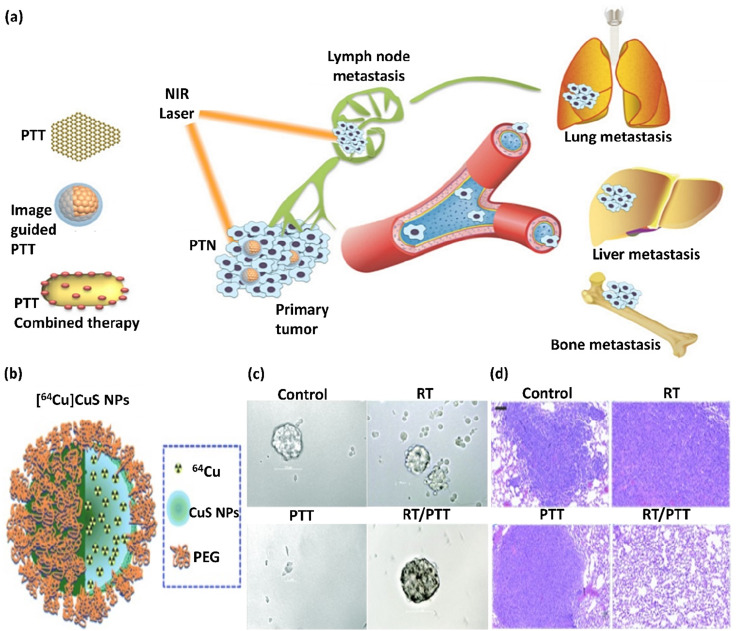
(a) Illustration of the strategies of using PTAs, which are divided into three categories: PTT alone, PTT with imaging guidance, and PTT combined treatment. This figure was adapted with permission from Ivy Spring, 2016, **6**(6), 762–772.^57^ (b) Schematic illustration of the structure of [^64^Cu]CuS NP. (c) Photographs of mammospheres formed from tumors in mice subjected to RT ([^64^Cu]CuS NPs without laser exposure), PTT (CuS NPs), and RT/PTT ([^64^Cu]CuS NPs) treatments. (d) RT, PTT, and RT/PTT treatment anti-metastasis impact against 4T1 breast tumor. Lung tissues from Balb/c mice with tumors were investigated. Bar: 100 μm. Radio-PTT performed by a single nano-sized platform of [^64^Cu]CuS NPs eliminated tumor-initiating cells and diminished lung metastasis. Fig. 2b–d were adapted with permission from *Nanoscale*, 2015, **7**(46), 19438–19447.^58^

Recent developments in nanomedicine have become extremely important in diagnosing and treating cancer. One of the promising achievements of nanoscience and nanomedicine is using nanomaterials in non-invasive photothermal therapy (PTT) for cancer treatment. Photothermal agents (PTAs) are substances used in PTT and can efficiently convert light into heat, resulting in the destruction of cancerous cells in a selective manner. Active nanomaterials in LSPR that can absorb near-infrared (NIR) light and convert it into heat are new PTAs used in PTT. A well-known example of photothermal nanotherapeutics (PTNs) is noble metal NPs.^56^ There are different strategies to destroy cancerous cells and reduce cancer metastasis using PTNs. These strategies, as depicted in Fig. 2a, are as follows: (1) direct ablation of cancer cells using PTA individually, where the photothermal agent is irradiated at the tumor site, and the resulting heat causes damage to cancerous cells. (2) PTT with imaging guidance. This strategy provides the possibility for the special and selective irradiation of the tumor or metastatic site and estimating and optimizing the irradiation time. (3) Efficient combined treatment using PTT with other treatment methods. The use of other treatments minimizes the possible difficulties of PTT administration, and with this strategy, the synergistic effect of different treatments and therapeutics can be investigated.^57^ Compared to traditional therapeutic approaches, PTT demonstrates specific advantages in treating cancer, such as enhanced specificity, minimum invasiveness, and accurate spatial–temporal selection. PTT and PTT combined treatment has been studied on animal specimens suffering from lung, bone, and lymph metastasis in many types of cancer, with therapeutic effectiveness. In this regard, Min Zhu *et al.* investigated a [^64^Cu]CuS nanocomposite (Fig. 2b) for the combination of PTT/radiotherapy, which significantly inhibited lung and breast metastasis in mice and considerably increased the animal survival, introducing a promising treatment for metastatic tumors. According to this study, [^64^Cu]CuS NPs in breast cancer PTT/radiotherapy eliminated the tumor-initiating cells. As shown in Fig. 2c and d, combining the PTT/radiotherapy approaches prohibited tumor growth and lengthened the life of the animals. The reduction in metastatic lung lumps (Fig. 2d) and mammospheres of tumor formation in the 4T1-induced metastatic breast cancer model (Fig. 2c) was executed through the [^64^Cu]CuS NPs-mediated combined treatment.^58^

After discovering the PPTT method, novel solutions were developed in 2003, focusing on the properties of the infrared spectrum and thermal treatment of tumors with NIR, in which noble metal NPs such as gold NPs played an essential role under magnetic resonance guidance.^59^ The NPs utilized in PTT as PTAs provide the possibility of tumor penetration because of their unique characteristics, such as high photothermal efficiency and small diameters. Thus, optimizing the structural features of NPs such as their shape, size, and composition can achieve unique optical, electronic, and mechanical characteristics in PTAs.^60^ Noble metal NPs have SPR properties, which increase the absorption of the radioactive and scattering features, making them efficient for PTT.^61^

Moreover, due to their high radioactive scattering characteristics, several methods, including PTT with imaging guidance, are accessible. In the PTT technique, a photosensitizer is excited with a unique wavelength. In contrast to PDT, PTT does not require oxygen for interaction with target cells or tissues. Recent research showed that light with a longer wavelength (lower frequency and lower energy) can be used in PTT, which causes less damage to normal cells and tissues.^62^ The PDT mechanism depends on the generation of photocatalytic reactive oxygen species (ROS), which changes the mitochondrial membrane potential and promotes apoptosis of cancer cells. The synthesis of novel photosensitizers was the main focus in early PDT research. For instance, several molecular systems containing porphyrin, chlorin, and phthalocyanine have been created as photosensitizers. In general, high-performance photosensitizers are anticipated to exhibit stronger long-wavelength absorption, a greater ROS quantum yield, reduced dark toxicity, and improved metabolic properties. Notably, there is considerable inconsistency among these criteria for high-performance photosensitizers. For instance, although long-wavelength light can penetrate denser tissues, its energy may be insufficient for the photocatalytic generation of ROS. However, the complex chemical synthesis required for the preparation of more effective photosensitizer molecules is likely to increase the cost of PDT.

Additionally, PDT has several restrictions. For instance, tumor tissue is often hypoxic and requires the appropriate quantity of oxygen. Besides, PDT has complicated impacts on the immune systems that fight cancer. Alternatively, given that tumors are less resistant to heat than normal tissues, PTT can be important cancer therapy, which depends on converting light into thermal energy for the thermal ablation of tumors by photothermal agents. The main advantages of PTT are its fewer side effects and low drug resistance. Also, different from photosensitizers, various materials can be used as photothermal reagents, including Au NRs, graphene, and MoS_2_ nanosheets.^63,64^

**Fig. 3:**
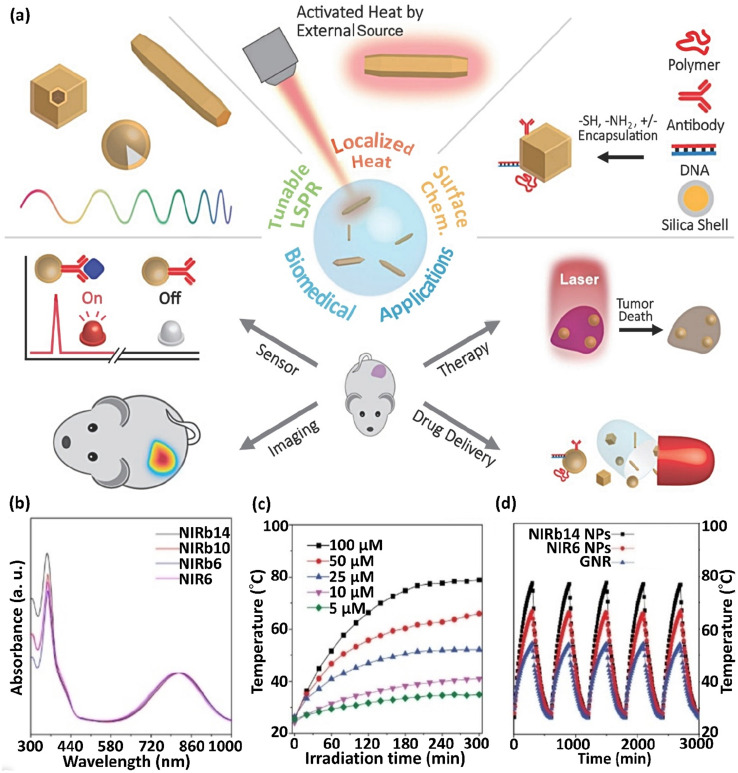
(a) Scheme showing the properties of photothermal NPs and their applicability in biomedicine. This figure was adapted with permission from *Advanced Science*, 2019, **6**(17), 1900471.^66^ NIRb14, NIRb10, NIRb6, and NIR6 molecules presenting molecular rotors and bulky alkyl chains grafted to the central donor–acceptor thiophene–thiadiazole moiety and (b) their normalized absorption spectra in THF. (c) Thermograms (*T vs.* time) of NIRb14 polymer NP at various concentrations (5–100 μM), *λ*_irr_ = 808 nm laser irradiation. (d) *T vs.* time in sequential irradiation/cooling runs, for NIRb14 and NIR6 N compared to AuNRs. These figures were adapted with permission from *Chemistry–A European Journal*, 2021, **27**(62), 15361–15374.^69^

Mainly, PPTT involves lesion preparation when transferring NPs to lesions using bioengineered carriers such as stem cells, microbubbles, and intravascular catheters. The detonation of NPs under NIR laser radiation results in their accumulation around the target molecule.^65^ In recent years, significant progress has been realized in the energy, catalyst chemistry, optics, biotechnology, and medicine of plasmonic NPs. In this case, their strong and adjustable optical response properties and the ability to change their photothermal impact *via* a light source are important in biomedicine.^66^ The main application of plasmonic nanomaterials is PTT.^67^ The optical features of plasmonic metal NPs depend on their size and shape, and the above-mentioned structural parameters influence their photothermal conversion efficacy (Fig. 3a).^68^ For example, the aggregation of molecular fluorophores into various NPs or their cores is a novel method to improve fluorescence, a phenomenon described as aggregation-induced emission (AIE).

Nevertheless, competitive thermal relaxation may be boosted in these arrangements and the emitted fluorescence and converted thermal energy may be balanced. Particularly, rigidifying the local peripheral promotes emission, whereas flexibility causes a complex charge transfer inside the molecules, improving their photothermal response. For example, molecular rotors and bulky alkyl chains grafted to a central donor–acceptor thiophene–thiadiazole section exhibited an absorption at ∼800 nm and enhanced photothermal molecular aggregation when separated as NPs in a poly(β-amino ester)-b-poly(caprolactone) polymeric shell (Fig. 3b–d). These NPs exhibited preferable thermal conversion compared to that of AuNRs and demonstrated remarkable stability in sequential irradiation/cooling runs (Fig. 3c and d). Similarly, the conjugation of tetraphenylethylene and naphthalene diimide-fused 2-(1,3-dithiol-2-ylidene)acetonitrile led to enhanced molar absorptivity and possessed robust complex charge transmittance in PEG-modified NPs, leading to a high photothermal effect (PTE). Specifically, combining AIE in luminogen-modified silica NPs with the thermal function of conjugated NPs demonstrated PTE as a newly defined method to obtain nano-theranostic arrangements for imaging-guided chemothermal and photothermal cancer treatment simultaneously. Single-walled CNTs (SWCNTs) are semiconductors. This characteristic relies on their diameter and the angle of chiral wrapping, leading to absorption in the range of 450–1600 nm based on their interband transmissions ascribed to van Hove singularities. The segregated SWCNTs exhibited intense fluoresce; however, in the case of their association in bundles, vibrational relaxation was preferable, with a great improvement in photothermal yield. Prussian blue (PB) is a coordinating polymer with alternating [Fe(CN)_6_]^4−^ centers (Fe^2+^) with Fe^3+^ ions that have octahedral coordination with the nitrogen atoms of the cyanide ligands. The absorption of PB NPs occurs at ∼700 nm, corresponding to the electronic charge transfer from the Fe^2+^ to Fe^3+^ centers, followed by non-emissive relaxation.^69^

In the case of AuNPs, their disadvantages such as long retention time, cytotoxicity, and low amount of targeted cancer cells limit their application as PTTs. Nevertheless, with the development of technology for chemical synthesis, AuNPs possessing various shapes and sizes, as well as desired properties can be synthesized, which can achieve multimodal cancer therapy with increased antitumor impact. There are various types of AuNPs, including AuNRs, nanoshells, nanospheres, nanocages, and nanostars. Thus, due to the numerous characteristics of AuNPs and lasers, the plausible cell death mechanism induced by NIR lasers is also diverse, influencing the anticancer impacts and PTT results.^70^

NIR light, the most commonly used laser for PTT, is absorbed and scattered less in tissue, resulting in more profound tissue penetration. Near-infrared light refers to light with wavelengths in the range of 750 and 1350 nm (biological window), which can be categorized into the first NIR window (NIR-I) with a wavelength in the range of 750–1000 nm and a second NIR window (NIR-II) with a wavelength range in the range of 1000–1350 nm. Several PTTs concentrate on NIR-I but have a shorter penetration depth in tissue. Conversely, NIR-II is more advantageous in PTT due to the deeper tissue penetration of light in the NIR-II range and enhanced maximum permissible exposure. In addition, living cells and tissues are not damaged by low-intensity NIR lasers based on their low absorption in the NIR region. Considering these advantages, NIR photothermal substances have wide applications, including therapeutics in cancer. Therefore, NIR photothermal substances can be used as antimicrobial, imaging, stimuli-responsive drug delivery, and cancer therapy agents.^71^ In this case, the photothermal conversion efficacy is the key factor in PTT effectiveness.

**Fig. 4:**
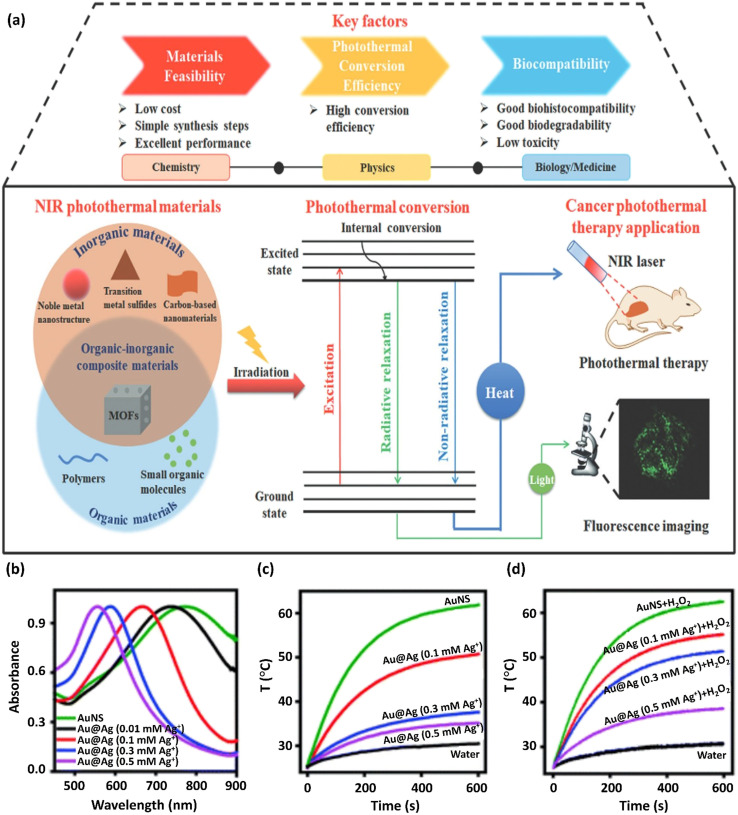
(a) Schematic representation of the photothermal conversion process and its applicability in photothermal treatment. This figure was adapted with permission from *Journal of Materials Chemistry B*, 2021, **9**, 7909–7926.^73^ The preparation and properties of Au@Ag@PEG/Apt with fine-adjusted plasmonic heating are represented. (b) UV-vis absorption spectra of AuNS upon the addition of various Ag^+^ concentrations to prepare Au@Ag nanostars. The normalization of spectra to unity are shown. (c) Curves of temperature change *versus* time for Au@Ag nanostars synthesized with various Ag^+^ concentrations on exposure to an 808 nm NIR laser at 1 W cm^−2^ power density. (d) Photothermal capability revitalization of Au@Ag nanostars by H_2_O_2_ (100 μM) etching of the coated Ag. The wavelength of the laser was 808 nm and the power density was 1 W cm^−2^. These figures were adapted with permission from *Chemical Science*, 2021, **12**(29), 10097–10105.^74^

When irradiating a photothermal substance with excitation light, certain procedures are initiated including absorption, scattering, and reflection. The absorbed light can be applied for heat generation by the above-mentioned processes. Hence, the PTT performance is proportional to the photothermal conversion efficacy and light absorption capacity of the photothermal substances. Based on electromagnetic radiation, photothermal substances can be divided into plasmonic localized heating metals and semiconducting non-radiative relaxation compounds, such as semiconducting polymers and small molecules. Advanced NIR-absorbed photothermal substances can be split into inorganic and organic substances and related composites. Among them, inorganic materials have the advantages of facile preparation, robust NIR absorption, convenient functionalization, and photostability.^72^ Also, organic and inorganic substances can be combined, forming organic–inorganic composites by chemical conjugation or physical affinity, providing functional property and photoelectric characteristics (Fig. 4a). Thus, certain disadvantages of common NIR photothermal substances, including single-function, poor photothermal conversion efficacy, and low biocompatibility, can be overcome through the incorporation of other materials.^73^ The plasmonic Au@Ag@PEG/Apt as a photo-agent prodrug exhibiting fine-adjusted plasmonic heating has recently been prepared.

The addition of excess amounts of Ag^+^ to a solution of Au nanostars (AuNS) increased their hydrodynamic size to 75.1 nm, resulting in the formation of Au@Ag nanostars. Furthermore, the LSPR of NS slowly shifted to the blue region by depositing more Ag ions (Fig. 4b) based on the Au and Ag plasmonic hybridization. A considerable reduction in the NIR absorption and a blue-shifted plasmon resonance peak was detected with an increase in the concentration of Ag^+^ from 0 to 0.5 mM (Fig. 4b), indicating the reduced photothermal conversion efficacy of Au substances on the nanoscale. Accordingly, upon exposure to NIR light irradiation, the increase in the NP solution temperature was inhibited given that more silver ions were diminished in AuNS (Fig. 4c). Firstly, the synthesis method was optimized to confirm the efficiency and safety of the photothermal factors in cancer treatment. The Au@Ag NS temperature with an Ag^+^ concentration of >0.1 mM was under the critical threshold of 43 °C for the destruction of cancerous cells. Alternatively, the H_2_O_2_ (100 μM)-etched Ag shell restored the astonishing heating capability of NSs (Fig. 4d).^74^

## 3. Brief comparison of LSPR-active materials

Noble metals, including Au and Ag, are the most used materials in the LSPR field. These noble metals exhibit LSPR in the visible range of the electromagnetic spectrum due to their d–d transition energy level. The resonant frequency of LSPR-active materials depends on not only their composition but also size, shape, dielectric environment, and particle–particle interaction and distance.^75^ The optical properties of plasmonic NPs and their therapeutic character make them specialized LSPR-active nanomaterials in theranostic applications.

AuNPs were the first materials employed to prepare plasmonic NPs *via* the reduction of a gold chloride aqueous solution with phosphorous.^76^ As an LSPR-active material, Au is a biocompatible metal with an inert nature, making it suitable for biological applications, especially considering that bioactive materials can be immobilized on the surface of AuNPs to construct specific targeting functionalized features. Furthermore, in the case of non-spherical AuNPs, they exhibit two dipolar resonance frequencies and two plasmonic bands in the UV-vis and near-infrared (NIR) regions.^77^

Silver is another crucial metal plasmonic NP ingredient. It is widely employed in medical applications due to its antibacterial and antimicrobial characteristics. Ag NPs have also been studied for their cytotoxic and genotoxic properties on several cancerous cell lines. Ag nanocubes with a size of 90 nm exhibited a strong resonance peak at 600 nm, whereas Ag nanospheres and shortened nanocubes of similar size exhibited peaks at 440 and 500 nm, respectively.^78^Table 1 displays comparative information about some AuNPs and AgNPs and the LSPR peaks of various shapes and sizes of NPs. Due to their biocompatibility, gold NPs are preferred over other nanomaterials with supreme properties. Alternatively, Ag NPs are considerably cytotoxic,^79^ promoting the use of less toxic and more biocompatible materials such as gold. However, the plasmon resonance strength of silver is greater than that of gold; therefore, silver exhibits higher surface-enhanced Raman scattering (ESRS) enhancement factors than gold.^80^ Compared to AuNPs with a size of 20 nm, which have an LSPR peak at around 520 nm, Ag NPs with the same size showed an LSPR peak at approximately 400 nm.^81^ Unlike AuNPs, Ag NPs are contaminated by atmospheric oxygen and sulfur reactions. Therefore, they need to be protected and enclosed by an inert and transparent shell in some cases.^82^ AuNPs with an almost analogous shape have similar LSPR peak positions. However, based on the diversity of their practical modifications, many of them will exhibit a red or blue shift.^83^

**Table 1:** LSPR preference of diverse Au and Ag NPs based on their shape and size

Nanostructure	Size (nm)	*λ*_LSPR_ (nm)	Light color	Reference
Ag nanospheres	42–45	439	Indigo	84
	15–40	420	Indigo	85
Au nanostars	28 (2–5 branches)	690	Red	86
Au nanospheres	50	530	Green	87
Au nanospheres	∼4	532	Green	88
Ag nanospheres	50	430	Violet	87
AuNRs	55.1 (±1.7) × 14.1 (±1.1)	800	NIR	89
Ag nanostars	100–120	380	Violet	90
Au aggregation nanospheres	50	700	Red	91
Au nanostars	40	730	Red	91
Ag nanospheres	40	530	Green	92
Au nanoshells	205.8 (±13.1) × 112.0 (±4.8)	808	NIR	93
Au nanoshells	151.1	∼850	NIR	94
Au nanocages	35 ± 3	532	Green	95
Au nanocages	∼60	790	NIR	96

NPs of some metals have LSPR properties, and due to these characteristics, they can be used in various fields such as bioimaging and visualization, biosensors, chemotherapy, and PTT. Meanwhile, gold and Ag NPs are preferred over other metals because of their biocompatibility. In addition, the LSPR wavelength of Au and Ag NPs is mostly in the NIR range, making the application of these NPs favorable in the cancer treatment field. Moreover, Ag NPs have antibacterial properties. NPs of other metals also have plasmonic properties, but their plasmonic properties and other inherent properties are unsuitable in many therapeutic fields.

## 4. Classification of porous micro- and nano-materials based on porosity grade

Generally, LSPR materials can be classified in various ways. For instance, they can be divided into groups based on their synthesis approaches, allowing their LSPR to be adjusted based on the material, particle size, and shape. Another way to categorize LSPR materials is based on their application in various fields such as environment, energy, biology. In this case, the classification of LSPR materials according to their porosity grade is significant because the pore tunability is dependent on the material synthesis approaches, resulting in materials with diverse morphologies and sizes. Measuring the optical signal (scattering or extinction, absorption) is plausible at a single particle (snpLSPR), nanostructure spot (ensemble-ensLSPR) or as imaging spectroscopy from a full perspective (LSPRi). From another point of view, the shape, dimension, porosity grade, and geometry of a material play a pivotal role. As mentioned above, different types of porous materials, such as silica, MOFs, and carbon-based tubes, are suitable for application as porous substrates for Au/Ag NPs and improving their therapeutic properties. The interaction between plasmonic NPs and porous nanomaterials is different and depends on their structure. For instance, when AuNPs are combined with MOFs, they are physically bound and incorporated in MOFs. However, after the formation of the Au/MOF composite, electron interaction occurs, which increases the Fermi level of the MOF and improves its charge properties.

Additionally, porous silica is the most desirable host platform for plasmonic materials due to its high surface area to volume ratio, simple production, and ability to regulate pore size during creation. When AuNPs are incorporated in the holes of porous silica, its open architecture encourages their integration and its high surface area makes increases their accessibility. The incorporation of AuNPs in porous silica changes its optical properties, where photoluminescence is achieved with higher intensity. In this case, the AuNPs will fill the pores of the porous silica, leading to enhanced photoluminescence.^50,97^

### 4.1. Silica materials

Chemically and thermally, porous silica is a material that has a uniform size and pore distribution, absorption capacity, and high surface area.^98^ In these particles, altering the calcination conditions and other parameters can adjust their size and shape while controlling other factors during their preparation such as temperature, reaction time, silicate amount/silica source, and surfactant concentration adjustment. The pore size and uniformity of porous silica are its advantages.^99^ Recently, several novel and modified synthesis methods have emerged for the synthesis of porous silica particles. The most common porous silica particles with a pore size in the mesoporous range are Mobil Crystalline Materials-41 (MCM-41),^100^ Santa Barbara Amorphous (SBA-15),^101^ Hiroshima Mesoporous Material (HMM-33), Technical Delft University (TUD-1), folded sheets mesoporous materials (FSM-16), MCM-48, SBA-11, SBA-12, SBA-16, and KIT-5. Table 2 demonstrates the most popular types of porous silica and their characteristics. Among the different mesoporous silica materials, MCM-50, SBA-11, and SBA-12 are remarkable adsorbents and catalytic substrates. Porous silica is suitable as a catalytic support due to its inertness, multiple functions, stability in various solvents, and improved catalytic selectivity.^102^

**Table 2:** Different mesoporous silica nanoparticle (MSN) types and their features

MSN family	MSN type	Pore symmetry	Pore size (nm)	Pore volume (cm^3^ g^−1^)
M41S	MCM-41	2D hexagonal	1.5–8	>1.0
	MCM-48	3D cubic	2–5	>1.0
	MCM-50	Lamellar	2–5	>1.0
SBA	SBA-11	3D cubic	2.1–3.6	0.68
	SBA-12	3D hexagonal	3.1	0.83
	SBA-15	2D hexagonal	6–0	1.17
	SBA-16	Cubic	5–15	0.91
KIT	KIT-5	Cubic	9.3	0.45
COK	COK-12	Hexagonal	5.8	0.45
FDU	FDU-12	3D cubic	10–26	0.91

### 4.2. Carbon nanotubes

CNTs or buckytubes are cylindrical allotrope carbon molecules with high strength and special electrical, mechanical, and thermal features, which are suitable for various industrial applications. CNTs have a wide band gap, high melting point, improved tensile strength, and thermal conductivity.^103^ Based on the number of tubes in their structure, CNTs can be classified into three categories including single-walled CNTs (SWCNTs), double-walled CNTs (DWCNTs), and multi-walled CNTs (MWCNTs). SWCNTs can be prepared using a graphene sheet that is folded and has a diameter of 1 to 2 nm and length that can be altered. These compounds can form crystal-like structures by gathering as organized hexagonal bundles. DWCNTs consist of two concentric CNTs, where their inner tube is surrounded by an outer tube, making them more stable and stronger than SWCNTs.^104^ MWCNTs consist of several layers of graphene twisted together and a diameter of 2–50 nm, corresponding to the number of graphene tubes. These tubes have an inter-layer distance of around 0.34 nm.^105^

### 4.3. Metal–organic frameworks

MOFs are classified as organic–inorganic hybrid and crystalline porous materials, greatly enhancing the field of porous materials. Generally, the structure of their framework, pore environment, and function can be controlled by adjusting parameters such as the choice of metal nodes and organic linkers, reasonable design of their topological structures, and modification after synthesizing skeletons. Hence, compared to conventional porous materials, MOFs have unique benefits.^106^ Hierarchical porous materials have multi-level pores and integrate the strengths of micro/meso and macroporous materials, thus effectively solving mass transfer problems. In general, the pores of functional materials can be categorized as micropores (<2 nm), mesopores (2–50 nm), and macropores (>50 nm). In research on MOFs, pores with a diameter of greater than 2 nm are of special interest. Hierarchical porous MOFs (HP-MOFs) maintain the functionality of micropores, provide high surface area, and create mesopores/macropores throughout the microporous matrix. Finally, they can provide the required space for accommodating large-sized species and reduce diffusion barriers.^107^

## 5. A brief survey of the efficient Ag/Au-plasmonic porous cargoes and their preparation methods

Today, AuNPs are applied because of their unique features such as high surface area (which leads to an increase in drug-carrying capacity), biocompatibility, non-toxicity, easy and available synthesis methods, stability against oxidation and degradation in the body, and electrical and magnetism properties compared to other carriers. Thus, they are used as nanocarriers in drug delivery, especially in cancer treatment (due to their nano size, AuNPs can penetrate tumor vessels). Gold NPs, which also act as carriers, can selectively deliver cargo, drugs, and biological substances to a specific area in the biological system. Their non-radiative properties are also very effective in cancer PPTT. Therefore, their use in the future has been highly considered.^73^ In addition, in light-sensitive materials, the optical properties of Au and Ag NPs are used to eliminate tumor cells, and these light-based treatments include PPT, PDT, and PIT.^11^

A green method for the synthesis of Ag/Au core–shell NPs for use in biomedical applications and cancer treatment was reported. Briefly, 5.0 g of *A. nilotica* shell (supplied by Khartoum locals, Sudan) was first rinsed with distilled water to eliminate dust. Afterward, it was combined with 50.0 mL of boiled distilled water. Then, overnight incubation was carried out at 25 °C, followed by filtration with filter paper and kept at 40 °C until use. In the next step, 1.0 mM of AgNO_3_ and HAuCl_4_ aqueous solutions were prepared. In the next stage, 50.0 mL of AgNO_3_ solution was added to the extract solution (10.0 mL) and continuously stirred for 15 min at 25 °C. Subsequently, a solution was obtained with a light-brown color, indicating the reduction of silver ions to silver NPs. Then, an aqueous solution of HAuCl_4_ was added. After 30 min, the solution color changed to a very dark-purple color, indicating the formation of Ag@Au core–shell NPs.^108^

Another method for the synthesis of Ag/Au NPs was reported, where 2.0 mL of AuNPs aqueous solution (2300 μg mL^−1^), AgNO_3_ (0.8 mL), and *Rumex hymenosepalus* solution (0.8 mL, *Rumex hymenosepalus* extract was utilized as a reducing agent for the preparation of NPs) was placed in a sterile glass culture tube. The centrifugation of the final mixture was executed at 12 000 rpm for 1 h after being thoroughly dispersed for 3 h in an ultrasonic bath. The collected solids were re-dispersed in ultrapure water with the help of sonication to reach a concentration of 1000 μg mL^−1^.^109^

The surface coating of a Ag NP template with PVP acted as a barrier that prevented the growth of the covered nanostructure and as a structural support substance that prevented the collapse of the nanostructure. Consequently, according to the above-mentioned article, the formation of a cluster porous nanostructure was attained *via* a two-step process. Initially, the galvanic replacement reaction executed at reduced temperature caused the rapid deposition of AgCl on the surface of Ag on all the reaction sites to overcome the challenge of continuous replacement, leading to the local inhibition of subsequent galvanic replacement reactions. Then, by completing the substitution reaction, the residual Ag and AgCl etching by surplus hydrogen peroxide revealed incorporated spherical cluster nanostructures. Overall, Ag/Au NPs are metals with intrinsic bactericidal characteristics, which when used together, have enhanced synergistic effects. The large surface area, remarkable electron conductivity, considerable physical properties, improved permeability, and long-lasting effect of these NPs make them promising materials in the biomedical field for diagnostics and therapeutics. AuNPs are the safest and least toxic materials for drug delivery.^29^ Furthermore, they have special chemical and physical characteristics, including increased surface Raman scattering and optical, electrical, and conductive behavior, high thermal and chemical stability, and non-linear and catalytic activity. Consequently, these characteristics of Ag NPs have been employed in electronic and medical applications. In addition, Ag NPs are conventionally applied as antimicrobial species to prevent the growth of microbes such as fungi, viruses, and bacteria.^111^ However, Ag/Au NPs have some drawbacks including potential toxicity, poor drug-loading capacity, and low capacity for loading hydrophilic drugs. Notably, although their small size is beneficial in medicine, it is potentially harmful to human health. Alternatively, the superiorities of Ag/Au NPs are their facile and large-scale preparation, and long-term stability, and thus they can act effectively act as controlled drug delivery systems.^112^

## 6. Bench assessment of PPTT-efficiency of Ag/Au-plasmonic porous cargoes

**Fig. 5:**
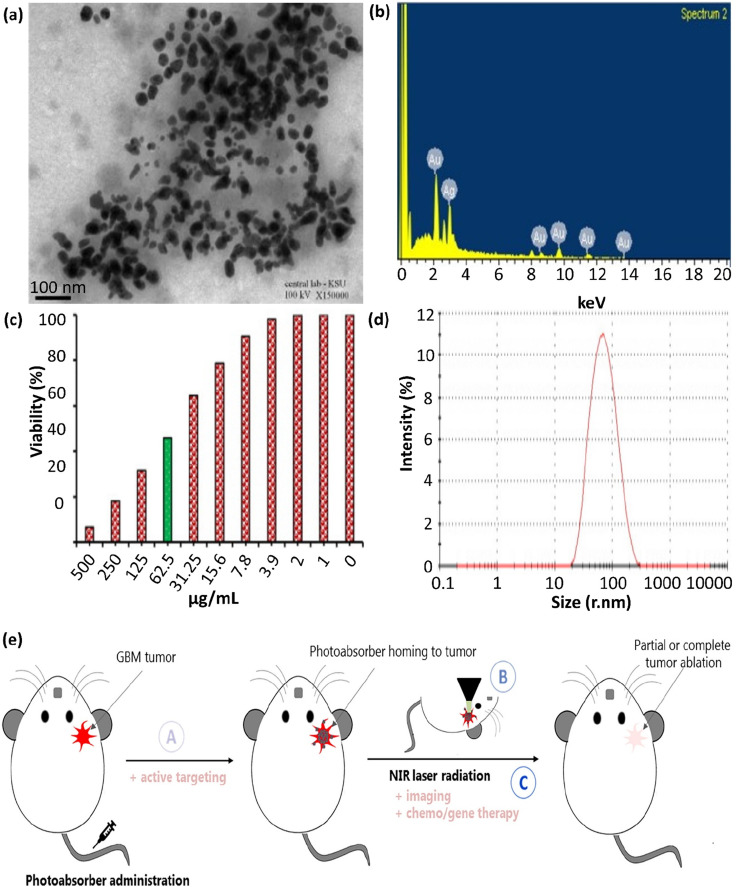
(a) TEM image of Ag@Au NPs, showing their partial agglomeration with spherical and some polygonal morphologies. (b) EDS spectrum, (c) MTT approach result corroborating the cytotoxic effect, and (d) DLS measurements of green Ag@Au core–shell NPs. These figures were adapted with permission from *Journal of King Saud University-Science*, 2022, **34**(4), 102000.^108^ (e) Illustration of the issues to address when improving PTT systems for GBM: (A) PTA agent has to pass through the BBB to access the tumor site; (B) *in situ* (and possibly in-depth) thermometry has to be located in the tissue site to monitor the increase in temperature inside the brain; and (C) the laser and PTA agent factors should be precisely placed and sufficient to induce localized HT at the tumor site, which ought to be profound in the brain. Active targeting, multimodal imaging, or chemo/gene therapy techniques can be combined to elevate the therapeutic impact of GBM PTT. This figure was adapted with permission from *Frontiers in Oncology*, 2021, **10**, 610356.^110^

In this section, the bench assessment of silver- and gold-plasmonic porous cargoes (Ag/Au-PPCs) in PPTT is assessed. Ag NPs were employed to determine the overall size of Ag/Au hollow nanoshells, where the addition of various volumes of 0.1% HAuCl_4_ modified the shell thickness. Ag/Au bimetallic hollow nanoshells were synthesized by adding various volumes of 0.1% HAuCl_4_ aqueous solution to equal amounts of Ag NPs. The catalytic performance of the Ag/Au bimetallic hollow and porous nanoshells (HPNSs) was first evaluated compared to Ag NPs (∼50–60 nm) and AuNPs (∼50 nm) with a similar size. By adding catalysts based on Ag or Au NPs, the absorption peak gradually decreased from 400 nm, indicating the reduction of the NO_2_ group in *p*-nitrophenol to NH_2_ (this reaction did not proceed in the absence of a catalyst). The porous structure of HPNSs was essential in reducing *r*-nitrophenol to *p*-aminophenol by NaBH_4_.^113^ In general, the plasmonic HPNSs synthesized in this project, due to their tunable optical properties, easy preparation, and specific hollow morphologies, can be used in different areas such as nanomedicine (cancer theranostics, drug delivery, *etc.*), bioassays, catalysts, and solar cells.^114^ The green synthesis of Ag@Au core–shell NPs (Fig. 5a) was reported using the medicinal plant *Acacia nilotica* in another study. An environmentally friendly, economical, non-toxic method was used, employing safe and non-hazardous materials. According to the results, *A. nilotica* is a regenerating and stabilizing agent for the preparation of NPs. Noble metal NPs, as previously mentioned, have unique optical and electronic properties, and according to Zhang *et al.*, they interact strongly with a specific wavelength. Bimetallic NPs demonstrate excellent absorption in the visible spectrum with the intensity of their peak in the range of 400–600 nm based on their SPR (SPR in the UV-visible spectrum occurs due to collective oscillations of conducting electrons).^108^

In addition, another study demonstrated the green preparation of Ag@Au core–shell NPs utilizing the husk extract of the *Acacia nilotica* plant. This green approach was non-toxic, environmentally friendly, convenient, economical, and utilized safe substances. The prepared Ag@Au NPs exhibited remarkably enhanced cytotoxicity against HeLa cancer cells at 500 μg mL^−1^ with IC_50_ values in the range of 74.6–7.43 μg mL^−1^, therefore showing potential applicability in cancer treatment, the pharmaceutical industry, and nanomedicine.^108^

The EDS analysis of Ag@Au core–shell NPs is shown in Fig. 5b. This spectrum indicates that the gold element exhibited absorption peaks at 2.15 keV and in the range of 8–14 keV, which are comparable to metallic gold NPs. Also, the significant absorption peak at 3.0 keV verified the presence of the silver element. The elements C and O_2_ were represented by the additional peaks at 0–0.5 keV. The findings from the EDS spectrum show that the strength of the gold and silver signals varied with the molar ratio of metal in the sample, and the absence of any other discernible peaks in the samples verified their high purity. Generally, these findings indicate that the solution contained a mixture of gold and silver atoms. Au acted as a shell around the Ag NPs with uniform sizes and morphologies.^108^ The anti-carcinogenic characteristics of the NPs in biological liquids were ascribed to their morphology, distribution, size, and structure. The anti-carcinogenic behavior of the prepared Ag@Au NPs was evaluated in a cervical carcinoma (HeLa) cell line. Based on the MTT assay (Fig. 5c), the growth of the HeLa cells was inhibited by 6.31% ± 1.36% at a concentration of 500.0 μg mL^−1^, showing an IC_50_ value of 74.6 ± 7.43 μg mL^−1^. Given that the size of the prepared NPs and their distribution are important in cancer treatment, the DLS measurement graph, as provided in Fig. 5d, confirmed the homogeneity and uniformity of the main quasi-spherical and few polygonal and quasi-rod morphology of the Ag@Au core–shell NPs.

In the next study, AuNPs and core–shell NPs (Au@Ag) were produced utilizing *Rumex hymenosepalus* root extract, a reducing agent, for the first time. To obtain Au@Ag NPs, a two-step sequential method was proposed, which formed particles with medium polydispersity and a homogeneous silver shell. For biological applications, it is important to obtain NPs with monodisperse sizes, and the average diameter of the Au@Ag NPs was found to be about 250 nm and remained constant as a function of concentration (the same approach was applied for monometallic NPs with an average diameter of about 122 and 135 nm for AuNPs and Ag NPs, respectively). Here, the PDI polydispersity index value was about 0.3 for monometallic NPs and 0.2 for Au@Ag NPs. Based on the results, which indicate that the size of the NPs was unaffected by their concentration, various NP systems exhibited desirable stability and an average dispersion size (0.3 ≥ PDI ≥ 0.2). In all cases, the NPs had a spherical morphology and were well separated from each other.^109^

## 7. Requirements for clinicalization of Ag/Au-plasmonic porous cargoes

### 7.1. Administration and dosage programming and measurement of irradiation

The use of small particles and molecular structures is increasing as a method for transporting drugs into the cells. The size of carriers is an essential feature for cell-penetration, and thus the properties of AuNPs can be altered to treat specific cancer-specific solid tumors. The PPTT utilized for superficial tumors (such as breast, head, neck, and melanoma) is restricted by the light penetration depth. In this case AuNPs, activated by the photothermal impact initiated with NIR light, are utilized more in PTT. NIR light has wavelengths in the range of 750–1700 nm (the first window is in the 750–1000 nm range and the second window is in the 1000–1700 nm range), where the water absorption is a minimal. This light can deeply penetrate tissues to reach the tumor area.^67^ Generally, cell death occurs *via* one of two distinguished paths, *i.e.*, (I) apoptosis and (II) necrosis. Apoptosis is reported to occur at 44 °C, whereas necrosis occurs at a temperature higher than 46 °C. In necrosis, the temperature disrupts the plasma membrane, causing the leakage of the cytoplasmic ingredients, and inflammation occurs.

Recent articles illustrated that PPTT can be designed to achieve apoptosis instead of necrosis through treatments.^115^ PPTT with high dosage (enhanced AuNP concentration, laser power, and exposure duration) results in necrosis whenever low-dose PPTT (low AuNP concentration, laser power, and exposure duration) can cause apoptosis.^116^ Regulating the laser exposure time and the size of AuNPs can be achieved to persuade cancer cells to undergo apoptosis *in vitro* and *in vivo* situations.^117^ In an experiment observing cancer cells (MCF-7 cells) or tumors (mammary gland tumors in dogs and cats) for 2 min, apoptosis dominantly occurred (42.7% of the population experienced apoptosis and 2.89% necrosis). Subsequently, 5 min laser irradiation (more than 500 times the dosage of 2 min irradiation) led 20.17% of the cells experiencing apoptosis and 15.5% undergoing necrosis, respectively. In this case, systematic analysis such as mass spectrometry (MS)-based proteomics is essential for more study on the mechanisms. It has been reported that AuNR-assisted PPTT in mice with head and neck tumors resulted in cytochrome c and p53-related apoptosis mechanisms.^67^

Intravenous injection of AuNPs is the main path for their entry in tumors. Also, they can be directly injected in tumors. Subsequently, AuNPs aggregate in the tumor based on their enhanced permeation and retention (EPR) effect, an event directly connected with immature and leaky tumor blood vessels. Besides, when the NPs are internalized in tumor cells, firstly, they have to pass through a barrier at enhanced interstitial fluid pressure and are surrounded by compact stromal tissues. In this case, smaller AuNPs may be more advantageous to overcome these barriers. The size of AuNPs in PPTT affects their biological behavior, where smaller particles are cleared from the kidney (<5 nm in size) and cross the blood–brain barrier (BBB, <20 nm). Alternatively, the majority of larger particles (>20 nm) are accumulated more in the liver and spleen. Smaller AuNPs in PPTT can enhance the heat generation efficiency, blood retention, and intratumoral penetration.^115,118–120^ Diverse target cell lines uptake plasmonic nanosystems *via* different paths based on the uptake time, size, shape, *etc.* (Table 3).^121–123^

**Table 3:** Summary of the PTT enhancement by plasmonic nanosystems considering the cell line, AuNP shape and size, laser type, and uptake time

Plasmonic nanosystem	Shape	Size (nm)	Laser type	Target cell	Uptake time	Ref.
CD44-antibody-PEG-AuNPs	Nanocages	58.4	CW 808 nm, 2.5 W cm^−2^	4T1 cells	24 h	124
Au-Ur@DTTC	Spherical	72.0	808 nm, 150 mW	SKOV3 and CT26 tumor	24 h	125
HAuNP@DTTC	Hollow sphere	85.0	808 nm	4T1 cells	24 h	126
MnO_2_@Au nanoenvelope	Spherical	150.0	808 nm, 0.25 W	PANC-1 and WI-38 cells	4 h	127
Ag@CuS NPs	Spherical	—	940 nm	HeLa cells	—	128
HA-4-ATP-AuNFs-DOX	Nanoframeworks	140.2 ± 3.2	1064 nm, NIR-II laser, 1 W cm^−2^	MDA-MB-231 breast cancer cells	2 h	129
AuNR-AS1411, AuNR-MUC1 (bioorthogonal SERS nanotags)	Nanorods	50.0 in length and 13.0 in width	808 nm, NIR laser, 0.2 W	MCF-7 breast cancer cells	4 h	130
Au@PB NPs	Spherical	72.0	808 nm laser, 2.0 W cm^−2^	4T1 mammary carcinoma cells	24 h	131
Nanoenvelope (ISQ@BSAAuNC@AuNR@DAC@DR5)	Nanorods	∼30.0 in length and ∼10.0 in width	808 nm	A375 human melanoma cells	—	132
Au nanostars (Au-4MBA-RGD)	Nanostars	295.4 ± 14.8	785 nm NIR-I laser, 390 mW cm^−2^, 1064 nm NIR-II laser, 1160 mW cm^−2^	A549 human lung adenocarcinoma cells	—	133
Aptamer-conjugated Au nanocage/SiO_2_	Regular cubic geometry	Outer edge length: ∼29, wall thickness: ∼3.5, slight corner truncation, and tiny pores on the side face	785 and 808 nm laser, 1.5 W cm^−2^	MCF-7 breast cancer cells	24 h	134
Au@Cu_2−*x*_S core–shell NPs	Hexagonal Cu_2−*x*_S (*x* = 1) (008) and face-centered cubic (fcc) Au	85.87 ± 10	808 nm laser, 0.45 W cm^−2^	HeLa cells and 4T1 cells	24 h	135
GNS-L/GB	Shells	85.0	808 nm laser, 4 W cm^−2^	MIA PaCa-2 and PANC-1 cells	48 h	136
Glu-AuNP	Sphere	16.0	X-radiation, 100 kVp, 10 Gy	MCF-7	2 h	137

Notably, brain tumor treatments have many challenges that need to be solved. For example, glioblastoma (GBM) is a highly aggressive primary virulent brain tumor, and the search for efficient therapies is an essential issue in pharmacy and a not fulfilled medicinal requirement. PPT enables the thermal destruction of tumors as a non-chemotherapy of GBM and circumvents the limitations of GBM heterogeneity, traditional drug resistance procedures, and side effects on circumferential health. Nonetheless, the development of this method is prevented by the peculiarities of tumors. Light-absorbing agents, such as NPs, must get to the tumor site at therapeutic concentrations and pass the BBB when administered systemically. Next, the NIR light illuminating the head has to overcome several hindrances to get to the tumor site with no local harm. The output intensity should be within safe limits, and the penetration depth must be adequate to induce profound, local HT and destroy the tumor. Monitoring the therapeutic process requires imaging methods that can precisely evaluate the temperature rise in the brain. As a non-chemical GBM therapy, PTT enables the thermal destruction of tumors, circumventing the heterogeneity of GBM, traditional drug-resistance procedures, and limiting side effects to healthy tissues in their vicinity, thus making it an attractive candidate for GBM treatment. Nevertheless, the peculiar properties of this type of tumor hinder its development, as presented in Fig. 5e.^110^

### 7.2. Biosafety assessments and excretion and degradation

The synthesis of AuNPs can be monitored by TEM, UV-vis, zeta potential, and DLS, which can be used to determine the size and uniformity of the synthesized AuNPs. *In vitro* photothermal effects were evaluated in studies, with two different concentrations (20 μg mL^−1^ and 100 μg mL^−1^) evaluated under 808 nm laser radiation for 3 and 5 min (2 W cm^−2^). After laser irradiation for 3 min, the temperature of the AuNR nanocomposite at 100 μg mL^−1^ increase to 51 °C, while that of NPs at 20 μg mL^−1^ was 46 °C. Ultimately, the temperature of the nanocomposite (100 μg mL^−1^) increased to 55 °C, while that of the NPs (20 μg mL^−1^) was 50 °C, demonstrating a dose–dependent relationship. All this evidence displayed the special photothermal impact of the NPs.^138^ Hence, it can be expressed that a huge scientific revolution has appeared with the introduction of AuNPs and their applications.^139–142^ The applicability of *in vivo* targeting-mediated substances with a nanometer scale typically involves the intravenous injection of these materials. Accordingly, the appropriate design of the size of nanomaterials is necessary to improve their bioavailability in the target sites. The efficient performance of nanomaterials should be considered as they travel from the injection site to the desired target through the bloodstream. This targeting relies on particle size for the following particular reasons. The colloidal stability of NPs is reduced with an increase in their size, where inorganic NPs of >100 nm and organic NPs of >500 nm typically exhibit poor dispersion stability. Therefore, the precipitation of large substances rendering low colloidal stability close to the injection size occurs, which cannot move further.

Moreover, fast excretion of the injected NPs through the kidney can be executed for NPs with a size of less than 5 nm. Importantly, this is because of the size limitation of the glomerular filter pores in the kidney with a size of 2–8 nm. Furthermore, the surface chemistry, biochemical stability, and particle shape of NPs affect their size. NPs with a rod or wire morphology have better targeting performance *in vivo* than similar-sized spheres due to their extended maintenance in the bloodstream by aligning it the bloodstream. Charged or toxic NPs induce immune responses for fast blood elimination through the reticuloendothelial system, namely, the liver and spleen. Therefore, PEG- or carbohydrate-surface-terminated nanomaterials have been prepared to reduce this clearance. Injected NPs with a size of <5 nm are quickly cleared by the kidneys, whereas NPs with a size of up to 100 nm aggregate in the lung, spleen, and liver.^143^ After irradiation with an 808 nm laser, the inhibition effect of AuNRs@PDA-PEG was improved by converting NIR light into heat. The red dots (dead cells) increased quickly in the image after the addition of AuNRs@PDA-PEG-DOX. After laser radiation, the percentage of dead cells was enhanced compared to the other groups, anticipating that the combination of PTT and chemotherapy can have a remarkable therapeutic impact in ablating tumors and inhibiting metastasis.^144^

The cytotoxicity was detected and authenticated *via* calcein AM/PI co-staining directly with NP treatment. The live and dead cells were determined utilizing the calcein AM/PI-containing approach, where the green color showed live cells and red indicated dead cells. Laser alone as a negative control showed a green color. PPTT without laser had a small number of cells with apoptosis. Alternatively, inhibition impact of the nanocarrier was ameliorated after irradiation with an 808 nm laser. The red dots (dead cells) increased, demonstrating that combining PTT and chemotherapy can have a significant therapeutic impact on the treated tumor and metastasis inhibition.^145^

The assessment of *Fritillaria cirrhosa* AuNPs was reported using a UV approach and SAED pattern help to monitor the size of AuNPs and uniformity during *in vivo* tests. The peaks in the selected area electron diffraction (SAED) pattern of the prepared AuNPs indicated that they were crystalline. The detected SAED ring pattern of the AuNPs demonstrated various planes, displaying the shape of the AuNP structures. Furthermore, their size, pattern, and dispersion were investigated by HR-TEM. The XRD analysis was carried out to support the SAED-TEM result. The diffraction peaks of the AuNPs at 2*θ* = 40.79° and 45.11° corresponded to the (1 1 1) and (2 0 0) planes, indicating a face-centered cubic structure. The peak intensity was proportional to the purity of the AuNPs. The results of the full-width at half maximum (FWHM) demonstrated the size of the prepared AuNPs. Based on body weight, kidney, and liver weight investigations, where the AuNPs were used to control the concentration of insulin, AuNP therapy eliminated muscle loss by preserving the normal insulin levels. The pathology of the pancreas was normal in the control rats, where the pathological alterations in the pancreas of the experimental and control groups were monitored by confocal microscopy.^146^ Also, confocal microscopy and photothermal images were employed to follow the AuNPs *in vitro* or *in vivo*.

Moreover, SERS spectra were recorded during apoptosis as a function of PPTT exposure time *in vitro*. In conclusion, the gentle condition of PPTT leads to the apoptotic path, which is desirable in cooperation with necrosis. In conclusion, AuNPs have more advantages with a size in the range of 20 to 200 nm; however, strategies to reduce their toxicity and renal clearance need to be studied.

## 8. Challenges and future perspective

Light-mediated therapies seem to be safer methods than conventional chemotherapies because no cytotoxic chemicals are used in these methods. However, similar to other newly developed strategies, there are challenges in the path of PPTT by plasmonic nanomaterials from the lab to the clinic. Firstly, the challenges in the excretion or degradation of the Au/Ag-based plasmonic cargoes in the internal environment of the body are discussed. Renal excretion depends on the cargo size; therefore, designing smaller species (*i.e.*, gold and silver dots) will be highly important. Also, it is crucial to note that the biodegradation of the cargo may cause high levels of toxicity in the blood serum. Therefore, the investigation of the toxicology and minimum effective dosage of Au/Ag-based plasmonic cargoes is very important. Indeed, the cargo should be designed to obtain optimized physiological stability and half-life (pharmacokinetics).

In most cases, the surface of the particles is conjugated to a biologically active agent (*e.g.*, Abs) to add a selective function in the targeted delivery of the cargo. In this case, the stability of the conjugation should also be optimized considering the type of attachment (covalent bonding or physicochemical interactions). As another potential limitation, the duration of the exposure of patients to a specific wavelength of light (maximum tolerance) should be considered because long durations may damage some vulnerable tissues or negatively affect the nervous system. The administrated dose of Au/Ag-based plasmonic cargoes may be a influential factor in the PPTT efficiency and exposure time. Considering all the above-mentioned matters, the design and development of new plasmonic cargoes based on Au and Ag dots (particle size <10 nm) can be listed at the top of plans in the future. Based on this suggestion, the challenges in the excretion and toxicity after the biodegradation of the particles will likely be addressed. In this case, the leakage of plasmonic dots from porous carriers should be precisely controlled because of the higher permeability of the pores to smaller particles. Different species of hybrid composites can be developed and utilized as carriers to deliver plasmonic Au and Ag dots to the target tissue to solve this problem. However, this field of science is developing rapidly, and many aspects of PPTT remain a hot topic to be investigated by researchers.

## 9. Conclusion

PTT of cancers using plasmonic nanomaterials has attracted significant attention because no cytotoxic drug is used during the treatment process. Therefore, the negative side effects after the treatment program are highly reduced *via* this method. Recently, the performance of gold and silver nanomaterials in PPTT was investigated by researchers. In this regard, many types of hybrid composites have been developed by the incorporation of Au and Ag nanomaterials in their structure for PPTT applications. These plasmonic nanomaterials exhibit the SPR effect. As a most used strategy, highly porous structures (*e.g.*, MOFs) were considered a host substrate for Au/Ag nanomaterials. The present survey attempted to provide an informative collection of the recent developments in the PPTT field, highlighting the potential challenges in the clinicalization of Au/Ag-based plasmonic cargoes. Herein, the routes for the preparation of Ag/Au-plasmonic porous cargoes and their related bench assessments were carefully reviewed. Moreover, the principal requirements for the clinicalization of Ag/Au-plasmonic porous cargoes were discussed. Finally, it was concluded that smaller species of Au/Ag plasmonic nanomaterials (namely gold and silver dots) may be the most suitable materials for PPTT because they induce convenient excretion and lower toxicity levels in the internal environment of the body.

## Data availability

The original raw data will be submitted upon receiving a request.

## Author contributions

R. T. L.: ideation, project design, conceptualization, writing of the introduction, supervision, and project administration; F. G.: writing the initial draft; S. Z. S.: writing the initial draft; R. D.: search and preparation of the sources; F. R. A.: search and preparation of the sources; A. E.; writing partial sections of the manuscript; Z. M.: search and preparation of the sources; Z. R.: graphics and software; A. K.: review; F. J.: writing partial sections of the initial draft; and edit; A. M.: co-supervision.

## Conflicts of interest

The authors declare no conflicts of interest.

## Supplementary Material
